# Are breast cancer patients with low distress at diagnosis at risk of psychological symptoms later in their disease trajectory? Considerations for when to screen for distress

**DOI:** 10.2340/1651-226X.2025.42367

**Published:** 2025-01-25

**Authors:** Rikke Langballe, Birgitte Mertz, Niels Kroman, Thomas Maltesen, Susanne Rosthøj, Pernille Envold Bidstrup

**Affiliations:** a Psychological Aspects of Cancer, Cancer Survivorship, The Danish Cancer Institute, Copenhagen, Denmark; b Department of Clinical Oncology and Palliative Care, Zealand University Hospital, Roskilde, Denmark; c Department of Breast Cancer Surgery, Rigshospitalet, Copenhagen, Denmark; d Statistics and Data Analysis, The Danish Cancer Institute, Copenhagen, Denmark

**Keywords:** Breast cancer, distress screening, psychological symptom trajectories, latent class analysis

## Abstract

**Introduction:**

To target psychological support to cancer patients most in need of support, screening for psychological distress has been advocated and, in some settings, also implemented. Still, no prior studies have examined the appropriate ‘dosage’ and whether screening for distress before cancer treatment may be sufficient or if further screenings during treatment are necessary. We examined the development in symptom trajectories for breast cancer patients with low distress before surgery and explored potential risk factors for developing burdensome symptoms at a later point in time.

**Methods:**

In total, 299 patients newly diagnosed with breast cancer who scored < 7 on the distress thermometer were included between August 2017 and October 2019 at the Department of Breast Surgery, Rigshospitalet, Copenhagen. Patients were followed through electronic questionnaires at baseline before surgery and after 6, 12, and 18 months. We used latent class mixed models to identify sub-groups of patients with similar development in distress, anxiety, depression, breast cancer-specific health-related quality of life, self-efficacy, and fear of recurrence over time. Logistic and multinomial regression analyses were applied to examine clinical and sociodemographic factors associated with specific symptom trajectories.

**Results:**

We did not identify any sub-groups of women with low distress at diagnosis who developed disabling psychological symptoms up to 18 months after diagnosis. However, we did identify a sub-group of 52% of the women who experienced persistent mild anxiety (Generalised Anxiety Disorder [GAD]-7 score 5–9). Adjusted for baseline treatment modalities and sociodemographic characteristics, women having low social support (odds ratio [OR]: 2.90; 95% confidence interval [CI]: 1.07–7.87) or living with a partner (OR: 3.18; 95% CI: 1.38–7.34) were more likely to experience persistent mild anxiety.

**Interpretation:**

The results show that the majority of women with low distress at breast cancer diagnosis do not experience an increase in psychological symptoms over time. Screening for distress at cancer diagnosis may be an essential step to identify most breast cancer patients in need of professional support for psychological symptoms.

## Background

Women with breast cancer may experience severe physical, psychological, and emotional symptoms during treatment and survivorship, which may strongly influence their quality of life [1, 2] as well as treatment adherence [3], potentially leading to poorer survival [4]. Studies have shown that 8–25% of breast cancer survivors may experience a persistent high symptom burden with impaired physical and social functioning from diagnosis and up to 3 years after [1, 2]. Systematically identifying vulnerable subgroups at high risk of burdensome persistent symptoms and reduced quality of life may be a way to target support and limit healthcare resources effectively [5]. Screening for distress in patients with cancer is recommended by the National Comprehensive Cancer Network [6] and is considered an essential part of the quality of cancer care across international cancer guidelines [5, 7]. As recommended by a Cochrane review on systematic screening for psychosocial well-being among patients with cancer [5], interventions such as Rehabilitation After Breast Cancer (REBECCA) have been developed to target breast cancer patients with high levels of distress at the time of diagnosis and to offer these patients support based on nurse navigation and regular screening for patient-reported outcomes (PROs) [8]. Breast cancer patients with high distress at diagnosis who were socially vulnerable in terms of less education, low social support, and low patient activation experienced stronger effects of the REBECCA intervention [8].

Still, screening for psychological symptoms in cancer patients is not yet part of standard cancer care in many countries [9], and a key knowledge gap lies in the limited evidence regarding the timing and frequency of screening [5]. Previous studies investigating symptom trajectories have shown a decrease or stability in distress, anxiety, and depression for breast cancer patients who reported low mean symptom scores at diagnosis up to 16 months after diagnosis [1, 10–14], while other studies showed delayed development of symptoms of depression up to 3 years after breast cancer treatment among a subgroup of patients (4 and 24%) [15, 16]. To better guide the management and care of breast cancer patients, it is essential to examine whether patients with low distress at diagnosis develop disabling symptoms and impaired quality of life at a later point in time. However, no previous study has examined if screening for distress before cancer treatment may be sufficient to identify the patients who need targeted psychosocial interventions or if further screenings after treatment are necessary. Moreover, a variety of sociodemographic and treatment-related factors related to high and chronic development in psychological symptoms have been identified [1, 10–12, 14, 16, 17]. Still, few studies reported factors associated with low or mild symptom development.

Using data from an observational cohort study including breast cancer patients who were screened with low distress at baseline as part of the REBECCA trial [8], we examined whether subgroups of patients developed high symptoms of distress, anxiety, depression, low levels of breast cancer-specific health-related quality of life (HRQol) and self-efficacy, and high levels of fear of recurrence during the 18-month follow-up. Furthermore, we explored whether treatment-related and sociodemographic factors at baseline increased the likelihood of specific symptom trajectories.

## Material and methods

### Study population

Between August 2017 and October 2019, newly diagnosed primary breast cancer patients (pre-surgery) at the Department of Breast Surgery, Rigshospitalet, Copenhagen, who met the following criteria – aged 18 years or older, Danish speaking, physically able to attend rehabilitation, and able to provide written informed consent – were invited to participate in the study. Exclusion criteria were severe cognitive problems or dementia and unmanaged psychiatric diseases that prevented participation [8]. Patients who experienced moderate to high psychological distress (score ≥ 7 on the Distress Thermometer) were invited to participate in the REBECCA trial [8]), whereas patients who had low psychological scores (score < 7 on the Distress Thermometer) were invited to a longitudinal electronic questionnaire study providing information before surgery (baseline) and at follow-ups after 6, 12, and 18 months.

### Risk factors

From the medical records, we obtained information on the following treatment modalities: chemotherapy (no; neoadjuvant; adjuvant), endocrine therapy (no; yes), Trastuzumab (no; yes), and radiotherapy (no; yes). From the questionnaires, we included self-reported information on education (< 12; 12–15; > 15 years), employment (employed; unemployed), cohabitating partner (no; yes), and social support measured using the short version of the Medical Outcomes Study Survey (MOS) with a higher score indicating better support (low < 25% quantile; high ≥ 25% quantile).

### Outcomes

All outcomes were collected from the questionnaire data and included distress measured using the Distress Thermometer with a score ranging from 0 to 10, with higher scores indicating higher distress. Anxiety was measured using the Generalised Anxiety Disorder (GAD-7) with a total score ranging from 0 to 21 and higher scores indicating higher anxiety, and a cut point of ≥ 10 indicating generalised anxiety. Symptoms of depression were measured using the Patient Health Questionnaire (PHQ-9) with total scores ranging from 0 to 27 with a higher score indicating a higher level of depression and a cut point of ≥ 10 indicating moderate to severe depression. Breast cancer-specific HRQol was measured using the Trial Outcome Index-Physical/Functional/Breast (TOI-PFB) score from the Functional Assessment of Cancer Therapy-Breast (FACT-B) scale with a total score ranging from 0 to 96, and with a higher score indicating higher HRQol. Self-efficacy was measured using the Patient Activation Measure (PAM) with a higher score indicating higher self-efficacy (low ≤ 67; high > 67). Fear of recurrence was measured using the Concerns About Recurrence Questionnaire (CARQ-4) with a higher score indicating a greater fear of recurrence and a cut point of ≥ 12 indicating clinical fear of recurrence.

## Statistical analyses

### Descriptive statistics

We present frequencies and percentages of treatment and sociodemographic factors at baseline to characterise the study population of women with low distress at diagnosis. For quantitative variables, we report the median and interquartile range (IQR) or mean and standard deviation (SD).

### Modelling approach

To identify patients who may require more comprehensive care during their disease course, we used a latent class approach as described by Proust-Lima et al. [18] and implemented in the R-package lcmm. This method allows for the identification of sub-groups (classes) of individuals with similar development over time for the outcomes of distress, anxiety, depression, breast cancer-specific HRQol, self-efficacy, and fear of recurrence, respectively.

### Transformation selection

We assumed that an underlying latent process, representing an unmeasured common factor, describes each trajectory. The latent processes were modelled using linear mixed models with time squared as fixed effects. To account for the correlation between measurements for each participant, correlated random intercepts and slopes on time and time squared were included in the models. Flexible nonlinear transformations were used to link the repeated outcomes to the latent processes [18]. This approach accounts for non-normal distributions and unequal interval scaling (curvilinearity). The best-fitting transformation was chosen from linear, beta, and spline transformations based on the Bayesian Information Criterion (BIC).

### Class determination

The number of classes needed to describe distinct patterns for each outcome was determined by fitting models with one to four different classes and choosing the optimal number based on BIC, ensuring each class contained at least 30 individuals (10%). Besides BIC, additional goodness of fit measures were considered for each model: Akaike Information Criterion (AIC), entropy (discriminatory power, values close to one indicating high discrimination), and ICL (Integrated Classification Likelihood criterion, balance between fit and discrimination).

### Classification assessment

After determining the optimal number of classes, the nonlinear transformation function, and the underlying latent processes, we assessed the model’s ability to classify individuals into latent classes. This was done by calculating the posterior probabilities of class membership. Each individual was assigned to the latent class for which they had the highest posterior probability. Good discrimination, that is high separation of classes, may be assumed when the mean class membership probability in each class exceeds 0.90.

### Analysis of risk factors and class membership

Once individuals were assigned to a latent class, we explored whether risk factors were associated with class membership using the two-stage method based on joint likelihood. This approach accounts for the uncertainty in class membership when estimating the associations between risk factors and class membership. These analyses were performed using logistic regression for models with two classes and multinomial logistic regression for models with three classes. The risk factors considered in these models are described above. The analyses were conducted in R version 4.3.3.

## Results

**Figure 1:**
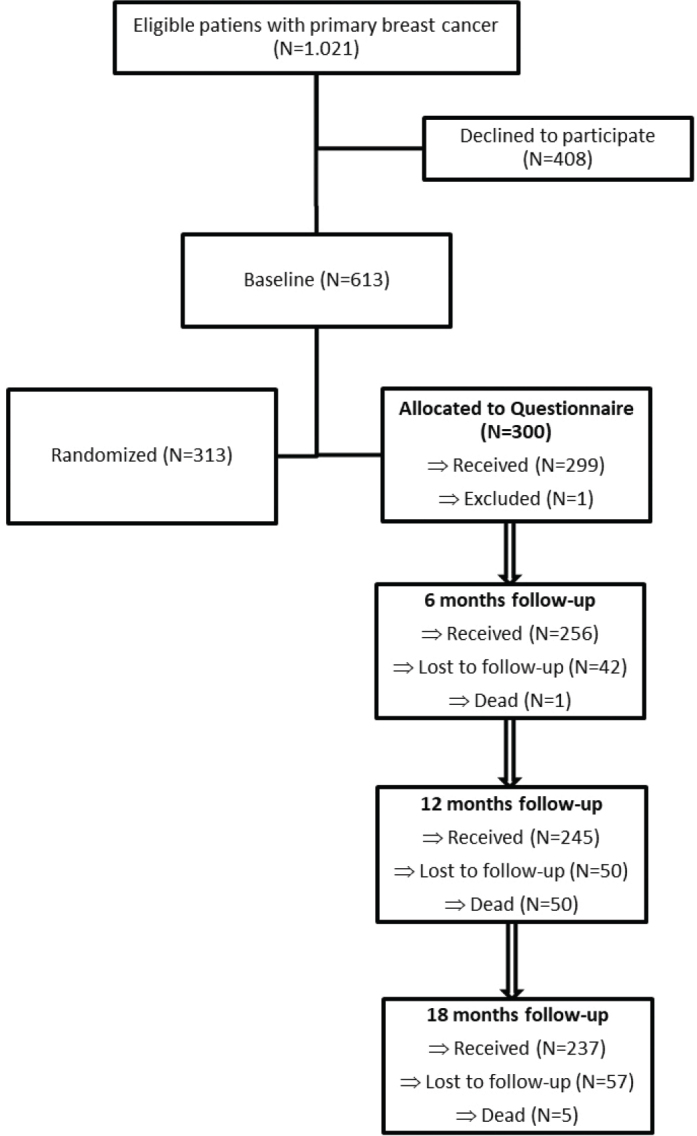
Participant flow diagram.

A total of 1.021 newly diagnosed breast cancer patients were eligible for the REBECCA trial and 60% agreed to participate (N=613). Of these, 313 patients with high distress entered the trial and 300 patients with low distress entered the observational study. One patient was subsequently excluded leaving 299 newly diagnosed breast cancer patients in the observational study (Figure 1). The women with breast cancer and low distress (mean score at diagnosis 3.2, SD 2.0) had a median age of 61.7 years (IQR 51.8–70, range 28.2–86.2), and the majority had a partner (60%); they were highly educated (66%) and more than half did not receive chemotherapy (Table 1). Development in distress, anxiety, depression, breast cancer-specific HRQoL, self-efficacy, and fear of recurrence showed an overall stable or decreasing pattern from diagnosis (baseline) and up to 18 months after (Supplementary Figure 1).

**Table 1:** Characteristics of 299 Danish patients newly diagnosed with breast cancer during 2017–2019 experiencing low distress before surgery.

Characteristics	Breast cancer cohort (*N* = 299)	
	No.	%
Age at baseline		
Median (IQR)	61.7	51.8–70
< 50 years	58	19
> 50 years	241	81
Cohabiting partner		
No	118	39
Yes	179	60
Missing	2	1
Social support		
Low (< 25% quantile)	63	21
High (≥ 25% quantile)	234	78
Missing	2	1
Education		
Short < 10 years	37	12
Medium 10–12 years	62	21
High > 12 years	196	66
Missing	4	1
Employment		
Not employed	152	51
Employed	144	48
Missing	3	1
Chemotherapy		
No	167	56
Adjuvant	88	29
Neoadjuvant	44	15
Endocrine therapy		
No	56	19
Yes	243	81
Trastuzumab		
No	267	89
Yes	32	11
Radiotherapy		
No	61	20
Yes	238	80

IQR: interquartile range.

### Symptom trajectories

**Figure 2:**
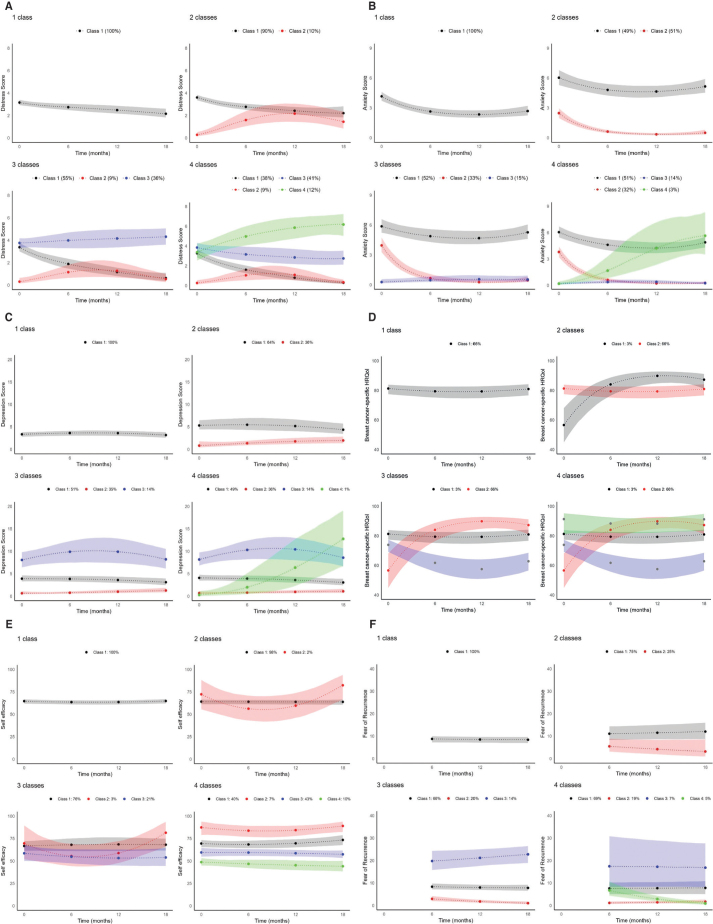
(A) Symptom trajectories of distress. (B) Symptom trajectories of anxiety. (C) Symptom trajectories of depression. (D) Symptom trajectories of breast cancer-specific HRQol. (E) Symptom trajectories of self-efficacy. (F) Symptom trajectories of fear of recurrence.

For distress, the model fit indices supported a two-group model (Table 2). Both sub-groups maintained no or mild distress throughout the 18 months of follow-up, with most women in group 2 (90%) following a decreasing pattern from a median distress score of 4 before surgery to approximately 2 and 3 during follow-up (Figure 2A).

**Table 2:** Model selection results for distress, anxiety, depression, breast cancer-specific HRQol, self-efficacy, and fear of recurrence for 299 women with newly diagnosed breast cancer.

Number of groups	Loglik	AIC	BIC	Entropy	ICL	Percent in each group (%)			
						Class 1	Class 2	Class 3	Class 4
Distress									
1	-1943.74	3913.476	3961.582	1	3961.582	100	NA	NA	NA
2	-1927.11	3888.229	3951.136	0.891809	3964.32	90	10	NA	NA
3	-1917.17	3876.334	3954.044	0.665882	4076.852	55	9	36	NA
4	-1910.78	3871.564	3964.075	0.656798	4116.004	38	9	41	12
Anxiety									
1	-2068.52	4163.034	4211.14	1	4211.14	100	NA	NA	NA
2	-2035.84	4105.684	4168.592	0.79845	4213.086	49	51	NA	NA
3	-2023.61	4089.221	4166.93	0.809932	4225.541	52	33	15	NA
4	-2005.74	4061.488	4153.999	0.874915	4201.631	51	32	14	3
Depression									
1	-2233.07	4492.137	4540.242	1	4540.242	100	NA	NA	NA
2	-2225.69	4485.371	4548.278	0.603968	4635.857	64	36	NA	NA
3	-2215.94	4473.872	4551.581	0.742277	4632.837	14	35	51	NA
4	-2208.78	4467.553	4560.064	0.786371	4644.487	49	1	14	36
BC-specific HRQol									
1	-3683.84	7393.682	7441.788	1	7441.788	100	NA	NA	NA
2	-3663.22	7360.433	7423.34	0.957065	7429.703	3	97	NA	NA
3	-3659.42	7360.835	7438.544	0.678772	7552.455	70	27	3	NA
4	-3658.28	7366.561	7459.072	0.667323	7601.671	66	3	28	3
Self-efficacy									
1	-3344.02	6714.04	6761.568	1	6761.568	100	NA	NA	NA
2	-3338.28	6710.56	6772.712	0.996836	6772.917	99	1	NA	NA
3	-3337.76	6717.524	6794.3	0.68366	6888.404	91	1	8	NA
4	-3336.2	6722.408	6813.807	0.674698	6941.138	71	1	21	8
Fear of recurrence									
1	-2194.48	4414.954	4461.972	1	4461.972	100	NA	NA	NA
2	-2188.63	4411.266	4472.751	0.861598	4493.343	96	4	NA	NA
3	-2179.21	4400.421	4476.373	0.804513	4532.847	64	16	20	NA
4	-2172.33	4394.655	4485.075	0.809521	4551.041	3	17	59	21

AIC: Akaike Information Criterion; BIC: Bayesian Information Criterion; BC: breast cancer; HRQoL: health-related quality of life; ICL: Integrated Classification Likelihood.

The model fit indices supported a three-group model for anxiety (Table 2). Approximately half (52%) of the breast cancer patients were classified with persistent mild anxiety (GAD score 5–9) throughout follow-up (group 1) (Figure 2B). The two other groups of women had minimal anxiety (score 0–4) from diagnosis and up to 18 months after which 33% of the women moved from a median anxiety score of 4 at diagnosis to a score of approximately 1 (group 2), and 15% remained at a score of 1 from diagnosis and up to 18 months after (group 3) (Figure 2B).

The model fit indices did not support multiple latent classes but instead favoured a one-group model for depression, breast cancer-specific HRQol, self-efficacy, and fear of recurrence (Table 2). As illustrated in Figure 2C–F, all trajectories indicated persistently mild psychological symptoms throughout the 18-month follow-up with median scores of approximately 4 for depression (PHQ-9), 75–80 for breast cancer-specific HRQol (TOI-PFB), 70 for self-efficacy (PAM), and 10 for fear of recurrence (CARQ-4).

### Risk factors associated with specific trajectories

Adjusted for treatment modalities and sociodemographic characteristics, women with short education were less likely to experience low levels of distress (group 1) as opposed to no distress (group 2) throughout follow-up (odds ratio [OR]: 0.19; 95% confidence interval [CI]:0.06–0.66) (Table 3). Women who had low social support and/or were living with a partner were more likely to experience persistently mild anxiety (group 1) compared with women with high social support and/or women living alone throughout follow-up (vs. no anxiety (group 3) (OR: 2.89; 95% CI: 1.06–7.86 and OR: 3.23; 95% CI: 1.40–7.49, respectively) (Table 3). No other apparent associations emerged for other baseline treatment modalities or sociodemographic characteristics and the development of distress and anxiety trajectories (Table 3). No ORs could be estimated for depression, breast cancer-specific symptoms, self-efficacy, and fear of recurrence as only one-group trajectory was supported.

**Table 3:** Odds ratios and 95% confidence intervals for associations between baseline treatment and sociodemographic characteristics and development of specific trajectories of distress and anxiety.

Baseline characteristics	Level	*N*	Distress (class 1 vs. 2)	Anxiety (class 1 vs. 3)	Anxiety (class 2 vs. 3)
			OR (95% CI)	OR (95% CI)	OR (95% CI)
Age (per decade)		290	1.09 (0.55–2.15)	0.71 (0.41–1.21)	0.72 (0.40–1.29)
Cohabitation	No	114	1.00	1.00	1.00
	Yes	176	1.70 (0.66–4.42)	3.23 (1.40–7.49)	1.85 (0.76–4.51)
Education	> 12 years	193	1.00	1.00	1.00
	10–12 years	60	0.44 (0.15–1.32)	0.78 (0.31–1.99)	0.90 (0.33–2.43)
	< 10 years	37	0.19 (0.06–0.66)	1.37 (0.40–4.73)	1.52 (0.40–5.69)
Employment	Employed	142	1.00	1.00	1.00
	Unemployed	148	0.71 (0.17–2.94)	0.52 (0.16–1.68)	1.19 (0.34–4.23)
Social support	High	231	1.00	1.00	1.00
	Low	59	3.10 (0.74–13.01)	2.89 (1.06–7.86)	0.64 (0.19–2.10)
Chemotherapy	Adjuvant	86	1.00	1.00	1.00
	Neoadjuvant	43	1.12 (0.18–7.04)	1.42 (0.26–7.84)	1.03 (0.16–6.82)
	None	161	0.75 (0.21–2.67)	1.67 (0.60–4.61)	0.96 (0.32–2.85)
Endocrine therapy	No	53	1.00	1.00	1.00
	Yes	237	2.53 (0.85–7.48)	0.84 (0.28–2.51)	0.74 (0.23–2.32)
Trastuzumab	No	258	1.00	1.00	1.00
	Yes	32	0.96 (0.18–5.17)	3.29 (0.53–20.34)	1.83 (0.26–12.90)
Radiotherapy	No	59	1.00	1.00	1.00
	Yes	231	0.44 (0.11–1.82)	0.60 (0.21–1.67)	0.58 (0.20–1.73)

OR: Odds ratio; CI: confidence interval.

## Discussion

In this observational cohort study of 299 breast cancer patients who were screened with low distress at diagnosis (< 7 score on the Distress Thermometer), we did not identify a sub-group of patients who experienced an adverse development in psychological symptoms (distress, anxiety, depression, breast cancer-specific HRQol, self-efficacy and fear of recurrence) throughout the 18-month follow-up. Instead, we identified sub-groups experiencing persistently mild or no psychological symptoms from diagnosis to 18 months after breast cancer treatment. Women with low social support or living with a partner were more likely to experience persistent mild anxiety (GAD-7 score 5–9), while women with short education were more likely to experience no distress during the first 18 months after their breast cancer diagnosis.

### Distress, anxiety, and depression trajectories

In agreement with our findings, previous cohort studies from Denmark [1, 11], China [14], Korea [12], and the United States (US) [10, 13] have shown a decrease or stability in distress, anxiety, and depression for breast cancer patients who reported low mean symptom scores at diagnosis and up to 16 months after diagnosis using group-based trajectory modelling. However, as most of these studies used different measures of depressive symptoms [1, 10, 11, 14], direct comparison of our results is difficult. Thus, distress, anxiety, and depression symptom trajectories among breast cancer patients with low distress early on remain scant and with various measures applied but suggest that in general, the symptom burden remains stable and low or decreases during the first 1.5 years after diagnosis. Possible mechanisms for this symptom pattern are complex and may include coping strategies such as positive reappraisal and problem-solving, as well as greater resilience and perceived support influencing the patients’ ability to adjust to a breast cancer diagnosis and to maintain a stable or increasing level of healthy psychological functioning [19–21]. Two recent cohort studies from 2022 and 2023, however, reported a subgroup of breast cancer patients who developed delayed depression symptoms 3 years after breast cancer treatment [15, 16]. Based on these previous findings, the development of long-term depressive symptoms beyond 1.5 years among women with low psychological symptoms at diagnosis should be explored further to evaluate whether additional screenings would be necessary.

### Health-related quality of life trajectories

Previous studies have demonstrated that HRQol on average improves over time for breast cancer survivors [22, 23]. However, previous studies examining distinct trajectories of HRQol using group-based trajectory modelling have identified a subgroup of breast cancer patients of 14–20% with persistently low HRQol from diagnosis and up to 1.5 years [24, 25]. The findings from these studies contrast with our study showing a stable and high HRQol among all patients. However, our study only included breast cancer patients who were screened with low distress levels at diagnosis, and who therefore may experience higher physical, emotional, and social functioning affecting the HRQol, which may explain the difference in findings. Overall, the previous findings as well as our study suggest that HRQol remains stable and high among breast cancer patients having high levels at diagnosis.

### Self-efficacy trajectories

We only identified one previous longitudinal study of 128 breast cancer patients using growth mixture modelling to describe self-management behaviours which is closely related to self-efficacy (Chinese version of the Chronic Disease Self-Management Questionnaire) up to 1 year from diagnosis [26]. The study identified two subgroups of women with the majority (65%) belonging to a subset with a sharp decreasing pattern who were characterised by being more anxious and having a low level of self-efficacy [26]. We identified only one trajectory with a stable and high self-efficacy using the PAM questionnaire throughout the first 1.5 years after breast cancer diagnosis among women who were screened with low distress at diagnosis, which is consistent with previous studies showing a positive association between high self-efficacy and less distress [27, 28].

### Fear of recurrence trajectories

Two previous studies from the US have examined trajectories of fear of recurrence among breast cancer patients using group-based modelling showing substantial variation in the development of fear of recurrence during 6 months to 5 years after surgery [29, 30]. These studies also showed that patients with low levels of anxiety (mean Hospital Anxiety and Depression Scale [HADS] score of 5.24) [30] and lower distress related to the initial diagnosis [29] had a low or stable fear of recurrence, which is in line with our findings of a stable and low fear of recurrence among women, who had low levels of distress at diagnosis.

### Factors associated with symptom trajectories

Our finding that low social support is associated with persistently mild anxiety agrees with previous studies showing an association between low support and high levels of anxiety and depression among breast cancer patients [16, 17]. Conversely, our finding that women living with a partner was related to persistently mild anxiety contrasts with previous studies showing that women without a partner were more likely to experience the most severe distress and depressive episodes [1, 10]. Contrary to our expectations, we found that women with short education were more likely to experience no distress during the first 18 months after their breast cancer diagnosis, which contrasts with previous studies showing that women with shorter education were at increased risk for chronic distress and depression [1, 11]. A potential explanation of our finding could be that women with shorter education levels may be less proactive in seeking health information including cancer treatment details, which may lead to less awareness of potential risks or complications and thus a reduced psychological burden [31]. Overall, sociodemographic and treatment-related factors of women with low psychological symptom levels are still not fully understood.

### Considerations for when to screen for distress

Systematic screening for distress among cancer patients has been recommended internationally and implemented primarily in the US, Canada, Australia [7], and at one Comprehensive Cancer Centre in Europe [32] but not in Denmark. Guidelines recommend screening for distress at minimum once at a medical visit, for example, at diagnosis, beginning and ending treatments, recurrence or progression, and referrals should be made for distressed patients to psychosocial oncology specialists and interventions with subsequent follow-up screening [7]. Our study contributes new knowledge suggesting that early use of a brief screening instrument at diagnosis may be an important first step in identifying breast cancer patients in need of referral or psychological intervention. Still, to guide implementation in clinical practice locally, important next steps would be to describe a detailed distress management plan including key personnel facilitating the screening, the psychosocial care services offered at the hospital, and the available referral options to patients experiencing high levels of distress [33]. To increase the potential for successful implementation, it is equally important to determine and systematically employ effective support strategies, such as ongoing engagement meetings and training sessions [9], as well as ongoing documentation and evaluation of the uptake of screening procedures, psychosocial care services, and referrals [34].

## Strengths and limitations

The strengths of our study included high-quality information on treatment modalities from medical records and the use of validated scales to longitudinally measure distress, anxiety, depression, breast cancer-specific HRQoL, self-efficacy, and fear of recurrence. Some patients may experience long-term burdensome symptoms after primary treatment [35]. It is thus a strength that we were able to follow breast cancer patients until 18 months after their diagnosis.

Our study also had limitations, including wide CIs, which may reflect heterogeneity or uncertainty in the associations with the development of specific psychological symptoms. At each time point, up to 19% of patients did not respond to the questionnaires (Figure 1). If non-respondents differ systematically from respondents regarding their psychological symptoms, and if non-participation is not random given the information used in our models, the level of symptoms may be underestimated. This could also lead to misclassification, particularly among patients with low participation rates. Additionally, five patients died during follow-up, and for these patients, our models implicitly impute symptom data beyond their time of death, which may have affected the accuracy of symptom course estimates.

## Conclusion

Systematic screening for distress at the time of breast cancer diagnosis may be an essential step to identify the vast majority of breast cancer patients in need of comprehensive psychological support during the breast cancer pathway. Whether additional screenings for distress during the breast cancer pathway are necessary needs further validation. With a breast cancer survivor population that continues to grow and a healthcare system experiencing substantial shortages, personalised -psychological support is an important step moving forward.

## Supplementary Material

Are breast cancer patients with low distress at diagnosis at risk of psychological symptoms later in their disease trajectory? Considerations for when to screen for distress

## Data Availability

Due to the EU regulation, the General Data Policy Regulation, we cannot share data with external parties without the prior consent of the participants. Collaboration projects will, however, be possible by contacting the last author Pernille Envold Bidstrup: pernille@cancer.dk.
