# Supplementary material for: Are breast cancer patients with low distress at diagnosis at risk of psychological symptoms later in their disease trajectory? Considerations for when to screen for distress
Source: Acta Oncol. 2025 Jan 25;64:42367. doi: 10.2340/1651-226X.2025.42367 (PMC11788679; doi:10.2340/1651-226X.2025.42367)
Supplement: Are breast cancer patients with low distress at diagnosis at risk of psychological symptoms later in their disease trajectory? Considerations for when to screen for distress [file AO-64-42367-s1.pdf]

Supplementary material has been published as submitted. It has not been copyedited, or typeset by Acta Oncologica

Supplementary Figure 1. Distributions of distress, anxiety, depression, breast cancer-specific health-related quality of life, self-efficacy, and fear of recurrence among 299 breast cancer patients at the time of diagnosis (baseline) and after 6, 12, and 18 months

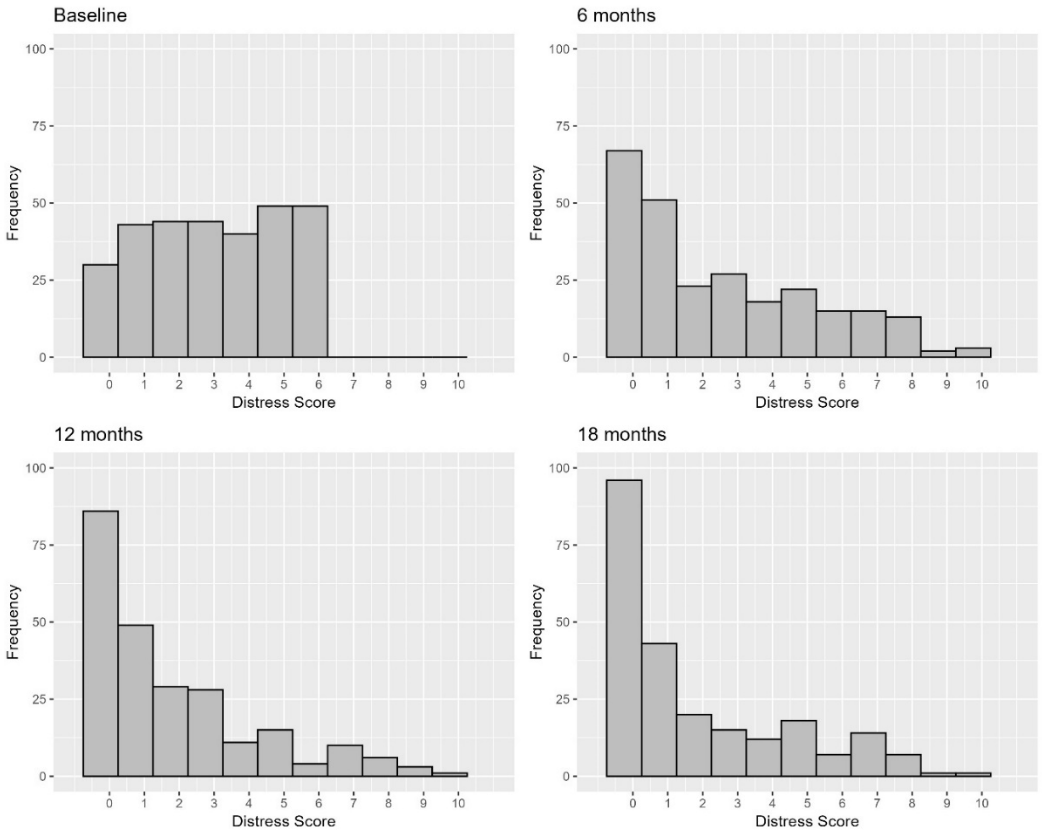

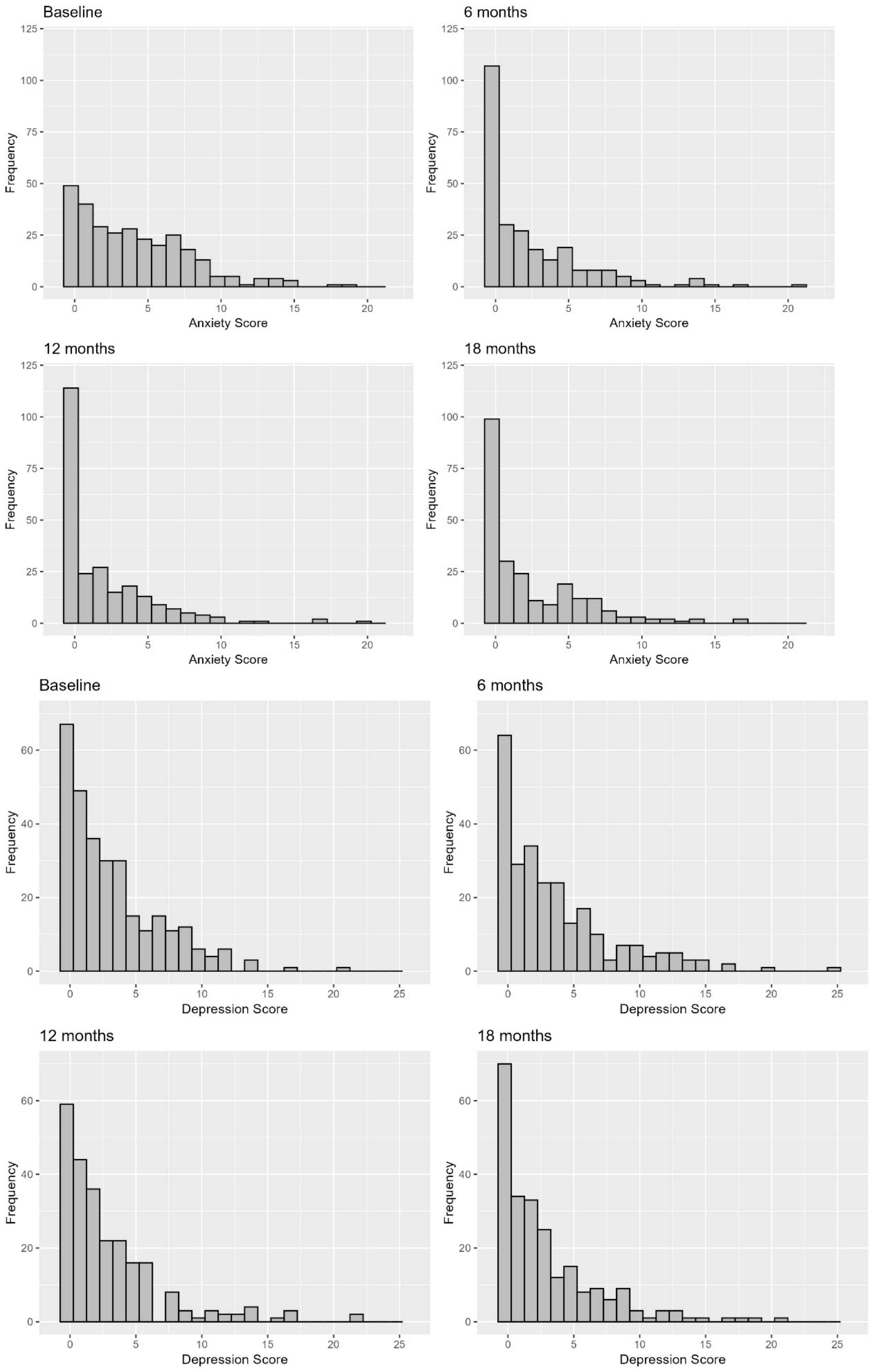
Baseline
6 months
12 months
18 months
Frequency
Anxiety Score
0
5
10
15
20
25
50
75
100
125
Baseline
6 months
12 months
18 months
Frequency
Depression Score
0
20
40
60
0
5
10
15
20
25

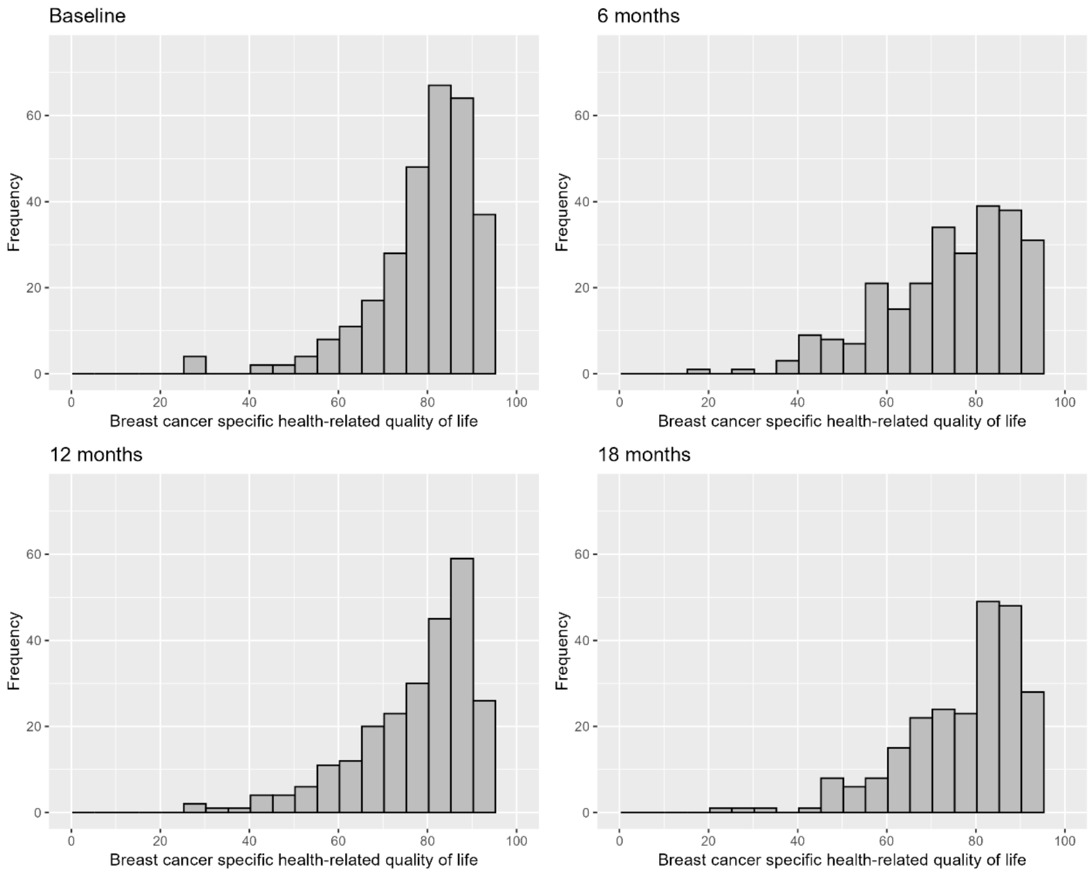
Baseline
6 months
12 months
18 months
Frequency
Breast cancer specific health-related quality of life
0
20
40
60
80
100

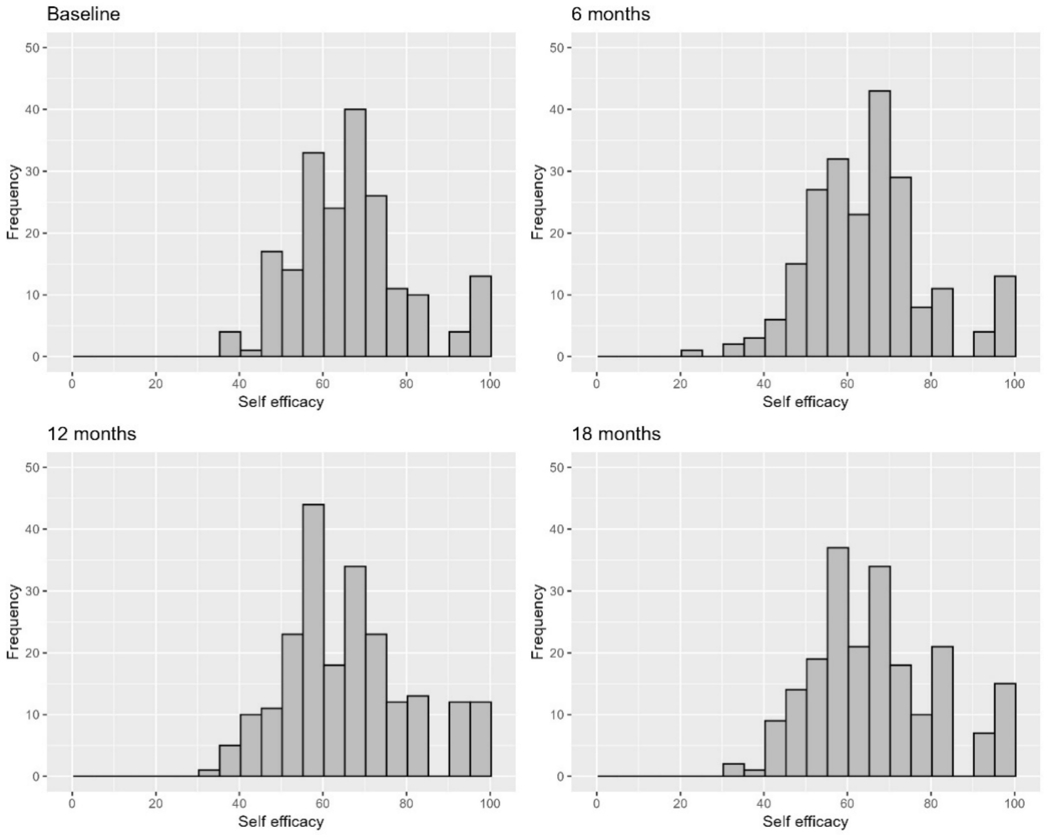
Baseline
6 months
12 months
18 months
Frequency
Self efficacy
0
10
20
30
40
50
60
80
100

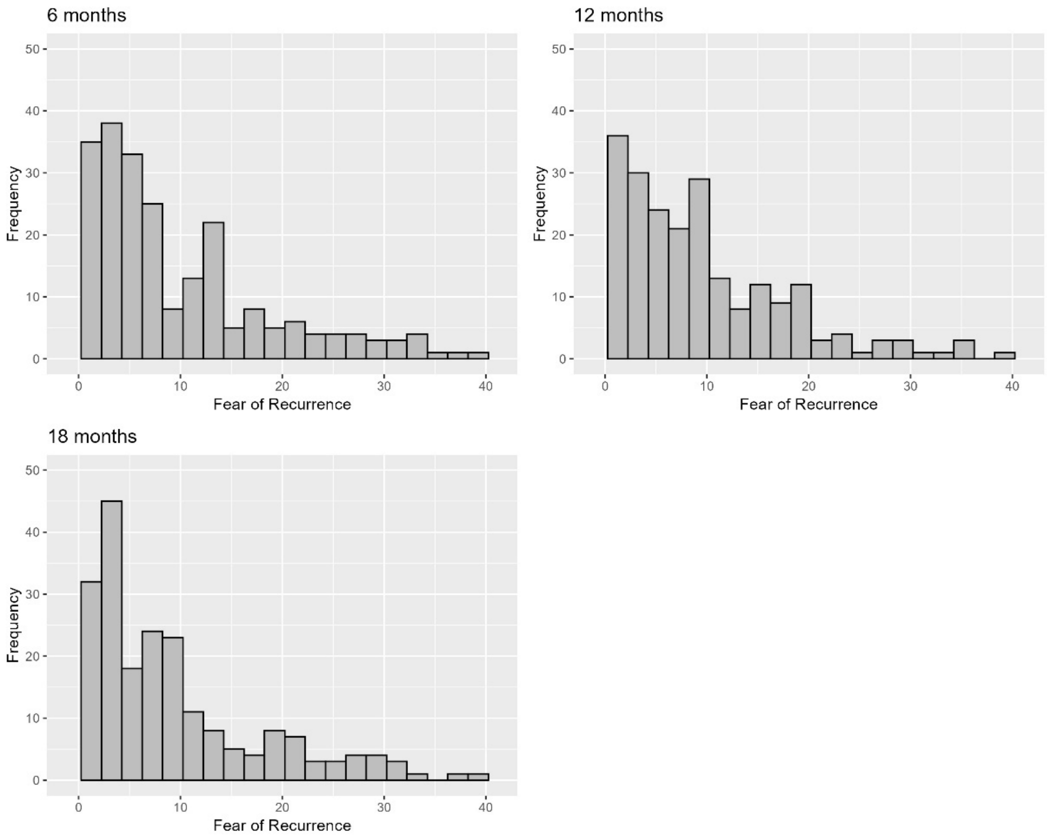
6 months
12 months
18 months
Frequency
Fear of Recurrence
0
10
20
30
40
50
